# Taxonomic and phylogenetic insights into dipteran-parasitizing *Ophiocordyceps*: Descriptions of two new species and a new record from China and Laos

**DOI:** 10.3897/mycokeys.127.176148

**Published:** 2026-01-23

**Authors:** Yong-dong Dai, Yu-hu Guan, Sheng-mei Wu, Shabana Bibi, Hui Chen, Chanhom Loinheuang, Jian-dong Liang, Yao Wang

**Affiliations:** 1 School of Basic Medical Science, Guizhou University of Traditional Chinese Medicine, Guiyang, Guizhou 550025, China School of Basic Medical Science, Guizhou University of Traditional Chinese Medicine Guizhou China; 2 State Key Laboratory of Discovery and Utilization of Functional Components in Traditional Chinese Medicine & School of Pharmaceutical Sciences, Guizhou Medical University, Guian New District, Guizhou 561113, China Guizhou Medical University Guizhou China; 3 Department of Biosciences, Shifa Tameer-e-Millat University, Islamabad 44000, Pakistan Shifa Tameer-e-Millat University Islamabad Pakistan; 4 Department of Biology, Faculty of Natural Sciences, National University of Laos, Vientiane 01080, Laos National University of Laos Vientiane Laos; 5 The High Efficacy Application of Natural Medicinal Resources Engineering Center of Guizhou Province, Guizhou Medical University, Guian New District, Guizhou 561113, China The High Efficacy Application of Natural Medicinal Resources Engineering Center of Guizhou Province, Guizhou Medical University Guizhou China

**Keywords:** Diptera, monophyly, multi-locus phylogeny, *
Ophiocordyceps
*, taxonomy

## Abstract

*Ophiocordyceps* species are renowned for their ecological roles and medicinal potential, yet their diversity on dipteran hosts remains insufficiently documented. Here, we investigated the diversity of dipteran-parasitizing *Ophiocordyceps* from China and Laos, describing two novel taxa—*O. calliphoridarum* and *O. laosensis*—and reporting *O. muscae* as a new record for Laos. Phylogenetic analyses based on a five-locus dataset (ITS, *nr*LSU, *tef-1α*, *rpb1*, and *rpb2*) strongly support the recognition of the two new species within the *O. dipterigena* complex of the hymenostilboid clade. *Ophiocordyceps calliphoridarum* is closely related to *O. muscidarum* but differs by parasitizing *Lucilia caesar* (Calliphoridae) rather than the housefly (Muscidae) and by possessing significantly larger asci and part-spores. *O. laosensis* closely resembles *O. muscae* but can be distinguished by its elongated perithecial ostioles and large asci and part-spores. Additionally, the asexual morph of *O. muscidarum* was newly described. These findings broaden our knowledge of the taxonomy and diversity of dipteran-parasitizing *Ophiocordyceps*, and further corroborate the phylogenetic monophyly of this lineage, thereby offering valuable insights into the co-evolutionary relationships between *Ophiocordyceps* fungi and their dipteran hosts.

## Introduction

The genus *Ophiocordyceps* was established by Petch in 1931 based on specimens parasitizing cockroaches with *O. blattae* as the type species. It is the most species-rich genus within the family Ophiocordycipitaceae, comprising more than 400 described species (http://www.indexfungorum.org) with a broad global distribution across tropical, subtropical, and temperate regions (Sung et al. 2007; Wijayawardene et al. 2017; Luangsa-ard et al. 2018; Araújo and Hughes 2019; Khonsanit et al. 2019; Wei et al. 2020; Dai et al. 2024; Xu et al. 2025; Guan et al. 2025).

Species of the genus *Ophiocordyceps* possess considerable value in medicine and biological control. The well-known Chinese caterpillar fungus *O. sinensis* (Berk.) G.H. Sung et al. has long been used in traditional medicine for its nutritional and therapeutic properties (Liang 2007; Dong et al. 2016). Other species such as *O. xuefengensis*, *O. liangshanensis* also exhibit notable antibacterial, antitumor, and antiviral activities (Yu et al. 2010; Wu et al. 2019). The *O. unilateralis* complex, known for manipulating the behavior of infected ants—the so-called “zombie-ant fungi”—provides a unique model for studying host specificity and parasitic manipulation (Evans et al. 2011; Will et al. 2020). Additionally, the *Hirsutella* anamorphs of *Ophiocordyceps*, such as *H. minnesotensis* and *H. rhossiliensis*, are effective biocontrol agents against nematodes and other agricultural pests (Rath 2000).

*Ophiocordyceps* species are frequently characterized by brightly and dull-colored stromata and perithecia that are either completely immersed or superficially distributed. Their ascospores are typically filiform, multi-septate, and often fragment into part-spores (Sung et al. 2007). Members of *Ophiocordyceps* exhibit an exceptionally broad host range, parasitizing insects from various orders, including Blattodea, Coleoptera, Diptera, Hemiptera, Hymenoptera, Lepidoptera, Mantodea, Neuroptera, Odonata, and Orthoptera. They are capable of infecting hosts at all developmental stages—larval (*Ophiocordyceps sinensis* etc.), pupal (*O. cochlidiicola* etc.) and adult (*O. nutans* etc.) (Sung et al. 2007; Shrestha et al. 2016; Luangsa-ard et al. 2018; Tasanathai et al. 2022; Tang et al. 2023; Dai et al. 2024; Mongkolsamrit et al. 2024; Chang et al. 2026).

Although *Ophiocordyceps* exhibits remarkable species diversity, with a wide range of host associations, species parasitizing adult dipteran insects remain relatively few. Considering the important role of dipteran pest control in agriculture and human activities, investigating the diversity of dipteran-parasitizing *Ophiocordyceps* species is of great significance. Dipteran insects serve crucial ecological roles in nature, yet certain species, such as fruit flies (Tephritidae), act as agricultural pests (Hudiwaku et al. 2021; Scolari et al. 2021), while others, notably mosquitoes, function as major disease vectors (Nainu et al. 2022). Additionally, some dipterans contribute beneficially to ecosystem regulation. Tachinid flies such as *Anagonia lasiophthalma*, *Exorista segregata*, and *Pentatomophaga latifascia* participate in natural pest control (Chen et al. 2020; Hammami et al. 2023; Martins et al. 2023). *Ophiocordyceps dipterigena*, initially reported by Berkeley and Broome (1873), represents the earliest known species parasitizing adult Diptera, characterized by light brown to brown stromata emerging from the host thorax and bearing sexual ascocarps at the stromatal tips. Advances in molecular techniques have since led to the discovery and updated taxonomic understanding of 10 *Ophiocordyceps* species associated with adult flies, which clustered to a well-supported monophyletic clade and referred to as the ‘*O. dipterigena*’ complex within the hymenostilboid clade (Mongkolsamrit et al. 2025; Yang et al. 2025).

This study investigates the diversity of *Ophiocordyceps* fungi associated with dipteran hosts. Through field surveys conducted in China and neighboring Laos, several dipteran-parasitizing *Ophiocordyceps* species were discovered. Detailed morphological examinations and multi-locus phylogenetic analyses were performed. We propose two new species and a new record of *Ophiocordyceps*, and provide supplementary taxonomic information for the known species *O. muscidarum*. Each taxon is described in detail with key diagnostic features, and the species diversity of dipteran-parasitizing *Ophiocordyceps* is further discussed.

## Materials and methods

### Specimen collection

Fungal specimens were collected from the forests of China and neighboring Laos. Habitat information, such as altitude and latitude, was recorded at the time of collection. Following in situ macrophotography to document the parasitic morphology, the specimens were promptly placed in dry plastic containers and transported to the laboratory for further examination. All specimens were dried and subsequently deposited in the Mycological Herbarium of Guizhou Medical University (**GMB**), China.

### Morphological study

Initially, macro-morphological features of the specimens were documented, with the color, and shape of the stromata, followed by observation under a dissecting microscope (Nikon SMZ745T, Nikon Corporation, Japan) to describe the fertile part and perithecia Sections of the fertile head were mounted on glass slides with a drop of lactic acid and lactophenol cotton blue, covered with a cover slip, and observed and photographed under a Leica DM2500 compound microscope (Leica Microsystems, Germany) for detailed measurements of perithecia, asci, peridium, apical cap, ascospores, and secondary ascospores. Asexual structures, comprising conidiogenous cells, phialides, and conidia, were observed on the surfaces of the host fly’s body and legs. The synnemata, typically arising from the host abdomen, were occasionally covered with *Hymenostilbe*-like phialides. The abdomen and legs were examined under a stereomicroscope to assess the presence of these phialides, and their morphological characteristics were recorded.

### DNA extraction, amplification, and sequencing

Genomic DNA was extracted directly from the wild specimen. Each specimen was thoroughly homogenized using a sterile rod, and total genomic DNA was then extracted using a commercial genomic DNA purification kit (Qiagen GmbH, Hilden, Germany). The quality-checked DNA was stored at –20 °C for subsequent analyses.

Polymerase chain reaction (PCR) was carried out in a 25 µL reaction volume, consisting of 1 µl DNA template, 1 µl each of forward and reverse primers (10 µM each), 9.5 µl ddH_2_O, and 12.5 µL of 2× Taq PCR Master Mix (TIANGEN, China). The following nuclear loci were amplified and sequenced: the internal transcribed spacer (ITS) region of ribosomal DNA, the translation elongation factor 1-α gene (*tef-1α*), and the genes encoding the largest and second largest subunits of RNA polymerase II (*rpb1* and *rpb2*). The primer pairs used were as follows: ITS5/ITS4 for ITS (White et al. 1990), LROR/LR5 for *nr*LSU (Vilgalys and Hester 1990), 983F/2218R for *tef-1α* (Rehner and Buckley 2005), CRPB1/RPB1Cr_oph for *rpb1* (Castlebury et al. 2004; Araújo et al. 2018), and fRPB2-5F2/fRPB2-7cR for *rpb2* (Liu et al. 1999; Sung et al. 2007). The amplification protocol consisted of an initial denaturation at 95 °C for 3 min, followed by 35 cycles of denaturation at 95 °C for 50 s, annealing at 55 °C for 1 min, and extension at 72 °C for 55 s, with a final extension at 72 °C for 10 min. PCR reactions were performed using a Bio-Rad T100 thermal cycler (Bio-Rad, USA). Amplified products were examined by electrophoresis on a 1% agarose gel stained with ethidium bromide in TBE buffer. The PCR products were subsequently purified and sequenced on the ABI3730XL automatic sequence analyzer (Sangong biotech, China).

### Host identification

Host identification was performed using a combination of morphological examination and molecular sequencing. For molecular identification, the mitochondrial *cox1* gene was amplified using primers LCO1490 and HCO2198 (Folmer et al. 1994). The PCR reaction system and thermal cycling program were the same as those used for the ITS sequences of the same samples. PCR products were sequenced using an ABI 3730XL automatic sequencer. The resulting sequences were analyzed using BLAST method to determine the host species.

### Phylogenetic analyses

Phylogenetic analyses were conducted using a combined dataset of five loci: ITS, *nr*LSU, *tef-1α*, *rpb1* and *rpb2*. Sequences newly generated in this study were combined with reference sequences obtained from previous publications and retrieved from GenBank (Table 1). Sequence alignment was performed with MEGA v6.06 (Tamura et al. 2013). The alignment parameters for ITS and *nr*LSU were set to default. For *tef-1α*, *rpb1*, and *rpb2*, only the exon regions were used. After alignment, sequences were examined in codon mode to ensure proper translation into proteins and to avoid the presence of stop codons or other errors caused by sequencing mistakes, with manual correction performed when necessary. *Paraisaria gracilis* and *P. phuwiangensis* were selected as the outgroup.

**Table 1.** List of taxa included in the phylogenetic analysis and their GenBank accession numbers.

**Species**	**Host/Substrate**	**Strain**	**Genbank accession number**					**Reference**
			ITS	*nr*LSU	*tef-1α*	*rpb1*	*rpb2*	
*Ophiocordyceps blattae*	Blattodea	BCC 38241	—	MT512657	MT533485	MT533479	—	Mongkolsamrit et al. 2021
*O. communis*	Blattodea, Termitidae	BCC 1842	MH754726	MH753680	MK284266	MK214110	MK214096	Tasanathai et al. 2019
*O. communis*	Blattodea, Termitidae	BCC 1874	MH754725	MH753679	MK284267	MK214109	MK214095	Tasanathai et al. 2019
*O. termiticola*	Blattodea, Termitidae	BCC 1920^T^	MH754724	MH753678	MK284265	MK214108	MK214094	Tasanathai et al. 2019
*O. termiticola*	Blattodea, Termitidae	BCC 1770	—	MH753677	MK284264	MK214107	MK214093	Tasanathai et al. 2019
*O. aphodii*	Coleoptera	ARSEF 5498	—	DQ518755	DQ522323	—	DQ522419	Spatafora et al. 2007
*O. appendiculata*	Coleoptera	NBRC 106959	JN943325	JN941412	AB968578	JN992463	AB968540	Ban et al. 2015
*O. brunneipunctata*	Coleoptera	OSC 128576	—	DQ518756	DQ522324	DQ522369	DQ522420	Spatafora et al. 2007
*O. curculionum*	Coleoptera	OSC 151910	—	KJ878885	—	KJ878999	—	Quandt et al. 2014
*O. houaynhangensis*	Coleoptera	MY11460	MH092892	MH092908	MH092899	—	—	Crous et al. 2018
*O. houaynhangensis*	Coleoptera	MY11461	MH092893	MH092909	MH092900	—	—	Crous et al. 2018
*O. annulata*	Coleoptera	CEM 303	—	—	KJ878962	KJ878995	—	Quandt et al. 2014
*Paraisaria phuwiangensis*	Coleoptera	TBRC 9709^T^	MK192015	MK192057	MK214082	MK214086	—	Mongkolsamrit et al. 2019
***O. calliphoridarum***	**Diptera (*Lucilia caesar*)**	**GMB 3129^T^**	**PX219623**	**PX225004**	**PX225635**	**—**	**PX245454**	**This study**
***O. calliphoridarum***	**Diptera (*Lucilia caesar*)**	**GMB 3130**	**PX219624**	**PX225005**	**PX225636**	**—**	**PX245455**	**This study**
***O. calliphoridarum***	**Diptera (*Lucilia caesar*)**	**GMB 3131**	**PX219625**	**PX225006**	**PX225637**	**—**	**PX245456**	**This study**
*O. dipterigena*	Diptera	OSC_151911	—	KJ878886	KJ878966	KJ879000	—	Quandt et al. 2014
*O. dipterigena*	Diptera	OSC 151912	—	KJ878887	KJ878967	KJ879001	—	Quandt et al. 2014
*O. dipterigena*	Diptera	HUA 186102	—	KJ917568	—	KF658664	KC610715	Sanjuan et al. 2015
*O. dipterigena*	Diptera	Hymdip995	—	KJ917573	—	—	KC610712	Sanjuan et al. 2015
*O. floriformis*	Diptera (*Clephydroneura* sp.)	BBH 27634	PV170894	OP493200	OP503163	—	OP503164	Mongkolsamrit et al. 2025
*O. floriformis*	Diptera (*Clephydroneura* sp.)	BBH 51295	PV170895	PV257643	PV274276	—	PV274287	Mongkolsamrit et al. 2025
*O. forquignonii*	Diptera	OSC 151908	—	KJ878889	—	KJ879003	KJ878947	Quandt et al. 2014
*O. forquignonii*	Diptera	OSC 151902	—	KJ878876	—	KJ878991	KJ878945	Quandt et al. 2014
*O. globiceps*	Diptera	MFLUCC 18-0495	MH725815	MH725829	MH727387	—	—	Xiao et al. 2019
*O. globiceps*	Diptera	MFLU 18-0661^T^	MH725816	MH725830	MH727388		—	Xiao et al. 2019
*O. hemisphaerica*	Diptera	FLOR 59525^T^	KX197233	—	—	—	—	Hyde et al. 2016
*O. hemisphaerica*	Diptera	FLOR 59542	KX197234	—	—	—	—	Hyde et al. 2016
***O. laosensis***	**Diptera (*Musca* sp.)**	**GMB 3137^T^**	**PX219630**	**PX225011**	**PX225642**	**PX225644**	**PX245461**	**This study**
***O. laosensis***	**Diptera (*Musca* sp.)**	**GMB 3138**	**PX219631**	**PX225012**	**PX225643**	**PX225645**	**PX245462**	**This study**
*O. muscae*	Diptera (*Musca domestica*)	BCC 73607	PV170896	PV257645	PV274277	—	PV274289	Mongkolsamrit et al. 2025
*O. muscae*	Diptera (*Musca domestica*)	BCC 73616^T^	PV170897	PV257646	PV274278	—	PV274290	Mongkolsamrit et al. 2025
***O. muscae***	**Diptera (*Musca* sp.)**	**GMB 3135**	**PX219628**	**PX225009**	**PX225640**	**—**	**PX245459**	**This study**
***O. muscae***	**Diptera (*Musca* sp.)**	**GMB 3136**	**PX219629**	**PX225010**	**PX225641**	**—**	**PX245460**	**This study**
*O. muscidarum*	Diptera (Muscidae)	HKAS 132178^T^	PQ423676	PQ423695	PQ675604	—	PQ569900	Yang et al. 2025
*O. muscidarum*	Diptera (Muscidae)	HKAS 132275	PQ423677	PQ423696	PQ675605	—	PQ569901	Yang et al. 2025
***O. muscidarum***	**Diptera (*Coenosia* sp.)**	**GMB 3132**	**PX219626**	**PX225007**	**PX225638**	**—**	**PX245457**	**This study**
***O. muscidarum***	**Diptera (Coenosia sp.)**	**GMB 3133**	**PX219627**	**PX225008**	**PX225639**	**—**	**PX245458**	**This study**
*O. philippinensis*	Diptera	LOD PF 4565^T^	OQ641807	OQ641808	OQ660303	—	—	Crous et al. 2023
*O. philippinensis*	Diptera	BCC 79225	PV170899	PV257648	PV274280	—	—	Mongkolsamrit et al. 2025
*O. philippinensis*	Diptera	BCC 78339	PV170900	PV257649	PV274281	—	—	Mongkolsamrit et al. 2025
*O. philippinensis*	Diptera	BCC 22048	PV170898	PV257647	PV274279	—	PV274291	Mongkolsamrit et al. 2025
*O. philippinensis*	Diptera	BCC 79871	—	—	PV274282	—	PV274292	Mongkolsamrit et al. 2025
*O. philippinensis*	Diptera	BCC 79872	—	PV257650	PV274283	—	PV274293	Mongkolsamrit et al. 2025
*O. tabani*	Diptera	BCC 45127	PV170901	PV257652	—	—	PV339938	Mongkolsamrit et al. 2025
*O. tabani*	Diptera	BCC 39918	—	PV257651	PV274284	—	—	Mongkolsamrit et al. 2025
*O. thilosuensis*	Diptera	BCC 46607	PV170903	PV257654	PV274286	—	—	Mongkolsamrit et al. 2025
*O. thilosuensis*	Diptera	BCC 47494	PV170902	PV257653	PV274285	—	PV274294	Mongkolsamrit et al. 2025
*O. anshunensis*	Hemiptera	GMBC 3026^T^	PP583071	PP577938	PP681121	PP681111	PP681116	Guan et al. 2025
*O. anshunensis*	Hemiptera	GMBC 3027	PP583072	PP577939	PP681122	PP681112	PP681117	Guan et al. 2025
*O. aphrophoridarum*	Hemiptera	MFLU 20-0641^T^	MW139322	MW139330	MW160163	MW160167	MW160165	Yang et al. 2021
*O. asiana*	Hemiptera	GMBC 3023	PP583068	PP577935	PP681118	PP681108	PP681113	Guan et al. 2025
*O. asiana*	Hemiptera	NBRC 101749	AB968408	JN941429	AB968589	JN992446	AB968550	Ban et al. 2015
*O. fulgoromorphila*	Hemiptera	HUA 186139^T^	—	KC610760	KC610729	KF658676	KC610719	Sanjuan et al. 2015
*O. fulgoromorphila*	Hemiptera	HUA 186142	—	KC610761	KC610730	KF658677	—	Sanjuan et al. 2015
*O. longissima*	Hemiptera	NBRC 106965	AB968406	AB968420	AB968584	—	AB968546	Ban et al. 2015
*O. neonutans*	Hemiptera	KEL113^T^	KX197239	—	—	—	—	Friedrich et al. 2018
*O. neonutans*	Hemiptera	KEL114	KX197241	—	—	—	—	Friedrich et al. 2018
*O. neonutans*	Hemiptera	KEL142	KX197244	—	—	—	—	Friedrich et al. 2018
*O. nutans*	Hemiptera	GMBC 3024	PP583069	PP577936	PP681119	PP681109	PP681114	Guan et al. 2025
*O. sobolifera*	Hemiptera	NBRC 106967	AB968409	AB968422	AB968590	—	AB968551	Ban et al. 2015
*O. sobolifera*	Hemiptera	TNS F18521	—	KJ878898	KJ878979	KJ879013	—	Quandt et al. 2014
*O. tessaratomidarum*	Hemiptera	MY10830^T^	—	MW280218	MW292434	—	—	Khao-ngam et al. 2021
*O. tessaratomidarum*	Hemiptera	GMBC 3025	PP583070	PP577937	PP681120	PP681110	PP681115	Guan et al. 2025
*O. tricentri*	Hemiptera	NBRC 106968	AB968410	AB968423	AB968593	—	AB968554	Ban et al. 2015
*O. yakusimensis*	Hemiptera	HMAS 199604	—	KJ878902	—	KJ879018	KJ878953	Quandt et al. 2014
*O. nutans*	Hemiptera	T70	AB366623	—	—	—	—	Sasaki et al. 2008
*O. asiana*	Hemiptera	BCC 84234^T^	MW285708	MW280201	MW292438	—	—	Khao-ngam et al. 2021
*O. australis*	Hymenoptera	HUA 186104	—	KC610763	KC610733	—	KC610713	Sanjuan et al. 2015
*O. australis*	Hymenoptera	Ophaus926	KF937350	KC610765	KC610735	KF658662	—	Sanjuan et al. 2015
*O. buquetii*	Hymenoptera	HMAS 199617	—	KJ878905	KJ878985	KJ879020	—	Quandt et al. 2014
*O. cylindrospora*	Hymenoptera	MFLU 17-1961	MG553635	MG553652	—	——	MG647029	Hyde et al. 2018
*O. evansii*	Hymenoptera	HUA 186159^T^	KP200889	KC610770	KC610736	MK863830	—	Sanjuan et al. 2015
*O. evansii*	Hymenoptera	HUA 186163	KP200890	KC610771	KC610737	MK863831	—	Sanjuan et al. 2015
*O. formicarum*	Hymenoptera	TNS F18565	—	KJ878888	KJ878968	KJ879002	KJ878946	Quandt et al. 2014
*O. granospora*	Hymenoptera	BCC 82255^T^	MH028143	MH028156	MH028183	MH028168	MH028177	Khonsanit et al. 2019
*O. granospora*	Hymenoptera	BCC 82256	MH028144	MH028157	—	MH028169	MH028178	Khonsanit et al. 2019
*O. irangiensis*	Hymenoptera	NBRC 101400	JN943335	JN941426	—	JN992449	—	Schoch et al. 2012
*O. irangiensis*	Hymenoptera	BCC 82795	MH028142	—	MH028186	MH028164	MH028174	Khonsanit et al. 2019
*O. khaoyaiensis*	Hymenoptera	BCC 82796^T^	MH028150	MH028153	MH028187	MH028165	MH028175	Khonsanit et al. 2019
*O. khaoyaiensis*	Hymenoptera	BCC 82797	MH028151	MH028154	MH028188	—	MH028176	Khonsanit et al. 2019
*O. lloydii*	Hymenoptera	OSC 151913	—	KJ878891	KJ878970	KJ879004	KJ878948	Quandt et al. 2014
*O. megacuculla*	Hymenoptera	BCC 82262	MH028146	MH028161	MH028191	MH028172	MH028180	Khonsanit et al. 2019
*O. megacuculla*	Hymenoptera	BCC 82984^T^	MH028148	MH028162	MH028192		MH028181	Khonsanit et al. 2019
*O. myrmecophila*	Hymenoptera	MFLU 16-2912	MF351726	MF372585	MF372759	—	—	Xiao et al. 2017
*O. pseudolloydii*	Hymenoptera	MFLU 22-0266	OQ127360	OQ127394	OQ186385	OQ186434	OQ186408	Wei et al. 2022
*O. sphecocephala*	Hymenoptera	NHJ4224	GU723778	—	GU797131	—	—	Luangsa-ard et al. 2011
*O. thanathonensis*	Hymenoptera	HKAS 102442	OQ127361	OQ127395	OQ186386	—	OQ186409	Wei et al. 2022
*O. thanathonensis*	Hymenoptera	MFLU 16-2910	MF850375	MF850377	MF872614	MF872616	—	Xiao et al. 2017
*O. vespulae*	Hymenoptera	GACP2017079^T^	—	MN044859	MN117076		MN107548	Long et al. 2021
*O. liangshanensis*	Lepidoptera	YFCC 8578	MT774249	MT774226	MT774247	MT774233	MT774240	Wang et al. 2021
*O. macroacicularis*	Lepidoptera	NBRC 100685^T^	AB968400	AB968416	AB968574	—	AB968536	Ban et al. 2015
*O. sinensis*	Lepidoptera	EFCC 7287	JN049854	EF468827	EF468767	EF468874	EF468924	Sung et al. 2007
*O. unituberculata*	Lepidoptera	YFCC HU1301^T^	KY923212	KY923212	KY923216	KY923218	KY923220	Wang et al. 2018
*Paraisaria gracilis*	Lepidoptera	GMBC 3066	PQ787761	PQ785779	PQ789222	PQ789225	PQ789228	Chen et al. 2025
*O. odonatae*	Odonata (Dragonfly)	TNS F18563	AB104725	KJ878877	—	—	—	Quandt et al. 2014

The final concatenated alignment had a total length of 4795 bp, with the following distribution across loci: ITS (863 bp), *nr*LSU (993 bp), *tef-1α* (1001 bp), *rpb1* (741 bp), and *rpb2* (1197 bp). This 5-gene supermix was further partitioned into 11 distinct segments: one segment each for ITS and *nr*LSU, along with nine additional segments corresponding to the three codon positions within the protein-coding genes *tef-1α*, *rpb1*, and *rpb2*. The optimal partitioning scheme and evolutionary models for the 11 predefined partitions were determined using PartitionFinder2 (Lanfear et al. 2017), employing a greedy algorithm and the Akaike information criterion. The analysis yielded the following 10 partitions: Partition 1—ITS:, Partition 2—*nr*LSU, Partitions 3–5—*tef-1α* codon1, codon 2 and codon 3, Partition 6—*rpb1* codon1, *rpb2* codon1; Partitions 7—*rpb1* codon2, *rpb2* codon2; Partitions 8—*rpb1* codon3, and Partition 9— *rpb2* codon3.

Phylogenetic analyses of the combined alignment were performed with RAxML-HPC BlackBox v8.2.12 (Stamatakis 2014) via the CIPRES Science Gateway with 1000 bootstrap replicates. Additional maximum likelihood (ML) analysis was carried out using IQ-TREE v2.1.3 (Minh et al. 2020) with ultrafast bootstrapping to estimate branch support. Bayesian inference (BI) method was performed using MrBayes v3.2.7a (Ronquist et al. 2012) for five million generations. The nucleotide substitution models for each partition in the three analytical methods mentioned above were automatically determined and output by PartitionFinder 2. After the analyses were completed, the bootstrap support values and posterior probabilities obtained from the three different methods were simultaneously annotated on the phylogenetic tree.

## Results

### Phylogenetic analyses

**Figure 1.**
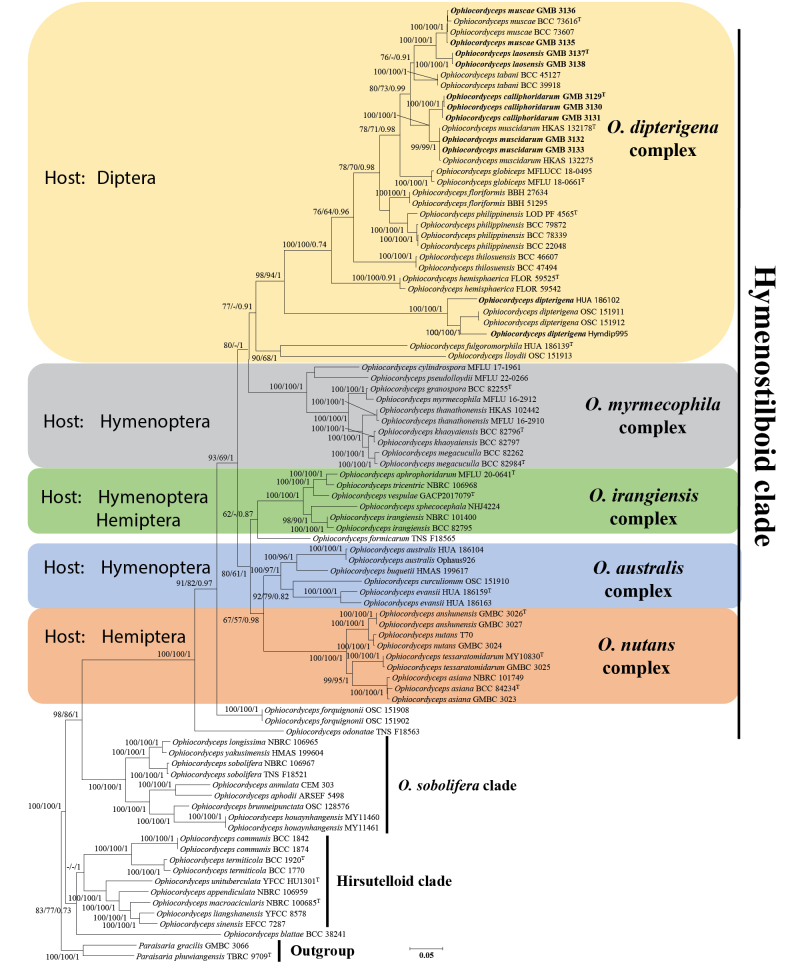
Phylogenetic relationships of *Ophiocordyceps* based on combined partial ITS + *nr*LSU + *tef-1α* + *rpb1* + *rpb2* sequences. Numbers at the branches indicate support values (IQ-TREE-BS/RAxML-BS/BI-PP) above 50%/50%/0.5. Ex-type materials are marked with “T”.

Fifty-four *Ophiocordyceps* taxa were included in the phylogenetic analyses conducted in this study. The dataset consisted of 99 samples in total, nine of which were newly generated. Phylogenetic trees reconstructed under both ML and BI criteria showed congruent topologies and supported the recognition of the previous well-defined clade —Hymenostilboid clade (Mongkolsamrit et al. 2025; Yang et al. 2025) (Fig. 1). This clade was mainly composed of five subclades, namely *O. dipterigena* complex (parasitizing flies, *Diptera*), *O. myrmecophila* complex (parasitizing ants, *Hymenoptera*), *O. irangiensis* complex (parasitizing wasps and spittlebugs, *Hymenoptera* and *Hemiptera*), *O. australis* complex (parasitizing ants and wasps, *Hymenoptera*), and *O. nutans* complex (parasitizing stink bugs, *Hemiptera*). This phylogenetic structure is highly consistent with previous reports (Mongkolsamrit et al. 2025; Yang et al. 2025).

The two new *Ophiocordyceps* species described in this study, together with 9 previously reported dipteran-parasitizing *Ophiocordyceps* species, were all clustered within the monophyletic group—‘*O. dipterigena*’ complex, which was defined by Mongkolsamrit et al. (2025) to represent *Ophiocordyceps* species parasitizing dipteran hosts. Two new species, *Ophiocordyceps calliphoridarum* and *O. laosensis*, were each resolved as distinct, well-supported lineages. *Ophiocordyceps calliphoridarum* formed a sister clade to *O. muscidarum*, whereas *O. laosensis* was recovered as sister to *O. muscae* (Fig. 1). The newly recorded *O. muscae* from Laos clustered within the same clade as conspecific samples, showing no detectable genetic divergence.

### Taxonomy

#### Ophiocordyceps calliphoridarum

Taxon classificationFungiOphiocordycipitaceae

Y. Wang & Y.D. Dai sp. nov.

2476FEF9-27E5-513D-8771-DD16A56F8A58

860783

Fig. 2

##### Etymology.

The epithet “calliphoridarum” refers to its host belonging to the family Calliphoridae (Diptera).

##### Holotype.

China, • Jilin Province, Dunhua City (43.41°N, 128.33°E, alt. 685 m), on *Lucilia caesar* (the species was identified by *cox1* sequence), on the trunk, 26^th^ Aug. 2024, collected by Kun Zhang, Yao Wang and Yongdong Dai (GMB 3129).

##### Description.

**Figure 2.**
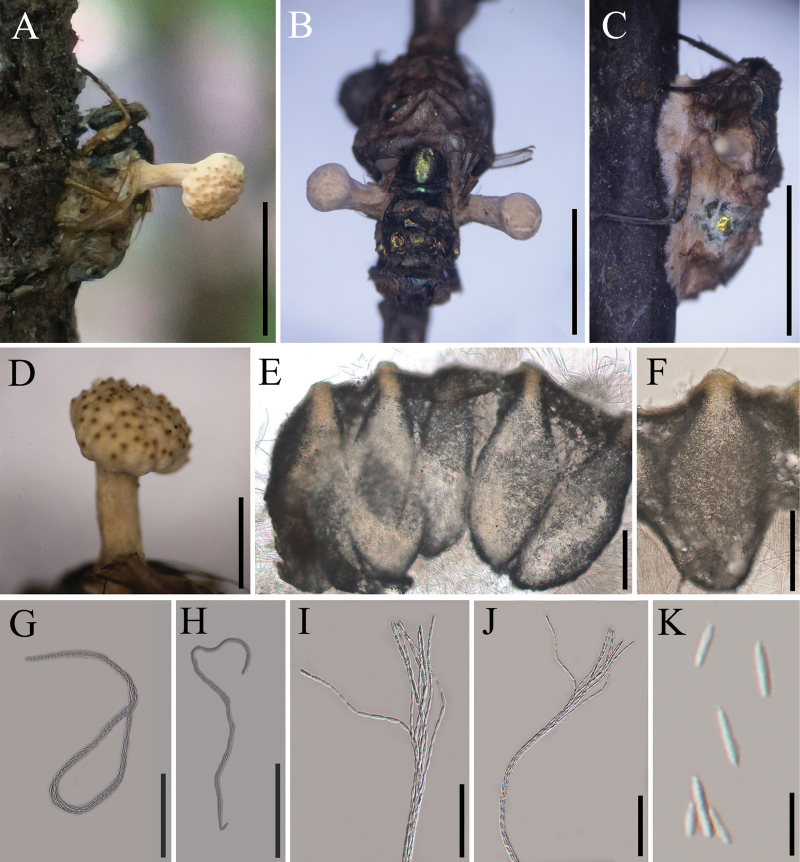
*Ophiocordyceps calliphoridarum*. **A, B**. Fungus on fly (*Lucilia caesar*, Calliphoridae, Diptera); **C**. The early-stage infected host by the *Ophiocordyceps* fungus; **D**. Stromata with spherical fertile part; **E, F**. Perithecia; **G, H**. Asci; **I–K**. Part-spores. Scale bars: 5 mm (**A–C**); 2 mm (**D**); 200 µm (**E, F**); 100 µm (**G, H**); 50 µm (**I, J**); 10 µm (**K**).

**Sexual morph**: ***Stromata*** stipitate, one or several arising from the prothorax and back region of the host, capitate, unbranched, pale yellow, 3–6 mm long, 0.5–1.5 mm wide with a fertile apex (Fig. 2A, B). ***Fertile heads*** hemispherical to globoid, upper surface slightly convex, moderate orange yellow, located at the terminal part of stipes, 0.5–1.5 mm thick, 1.5–3 mm diam (Fig. 2D). ***Perithecia*** 620–750 × 180–300 μm (x̄= 706 × 235 µm, n = 20), immersed, flask-shaped. ***Asci*** 230–510 × 6.1–8.3 μm (x̄ = 386 × 7.4 µm, n = 20), 8-spored, hyaline, cylindrical. ***Apical cap*** 2.6–4.5 × 3.8–5.6 μm (x̄ = 3.6 × 4.3 µm, n = 20), thick, hyaline. ***Ascospores*** filiform, multi-septate, breaking into part-spores, cylindrical to fusoid, 8.5–11.5 × 1.5–3.5 μm (x̄ = 9.8 × 2.6 µm, n = 60). **Asexual morph**: Not observed in natural substrates.

##### Host.

*Lucilia caesar* (Calliphoridae, Diptera).

##### Habitat.

The specimens were found on the trunk of a dicotyledonous plant.

##### Other material examined.

China. • Jilin Province, Dunhua City (43.36°N, 128.27°E, alt. 640 m), on *Lucilia caesar*, 28^th^ Aug 2024, collected by Kun Zhang, Yao Wang and Yongdong Dai (GMB 3130, GMB 3131).

##### Notes.

From a phylogenetic perspective, *Ophiocordyceps calliphoridarum* is closely related to *O. muscidarum*, yet it is distinguished by the formation of an independent clade with high statistical support (Fig. 1; 99/99/1.0). Both species parasitize dipteran hosts; however, *O. calliphoridarum* infects *Lucilia caesar* (Calliphoridae), whereas *O. muscidarum* targets the housefly (Muscidae). Micromorphological examinations further reveal that the asci and part-spores of *O. calliphoridarum* are significantly larger than those of *O. muscidarum* (Table 2). Based on integrated morphological and phylogenetic evidence, *Ophiocordyceps calliphoridarum* is proposed herein as a new taxonomic taxon.

**Table 2.** Summary of morphological comparison among *Ophiocordyceps calliphoridarum*, *O. laosensis* and related taxa.

Species	Host	Stromata (mm)	Distribution	Perithecia (μm)	Asci (μm)	Part-spores (µm)	References
***O. calliphoridarum***	**Diptera (Calliphoridae)**	**Single or Multiple, 3–6 × 0.5–1.5**	**China**	**flask-shaped, 620–750 × 180–300**	**Cylindrical, 230–510 × 6.1–8.3**	**Cylindrical, 8.5–11.5 × 1.5–3.5**	**This study**
***O. laosensis***	**Diptera (Muscidae)**	**Multiple, 5–9 × 0.5–1.3**	**Laos**	**flask-shaped, 320–1300 × 150–380**	**Cylindrical, 480–570 × 4.0–12.0**	**Cylindrical, 11–15 × 2.0–4.7**	**This study**
*O. muscae*	Diptera (Muscidae)	Multiple, 4–8 × 0.5–1.5	Thailand	Ovoid to obclavate, 820 –1100 × 320–400	Cylindrical, up to 720 long, 4–5 wide	Cylindrical to fusoid, 10–13 × 1.5–2	Mongkolsamrit et al. 2025
***O. muscae***	**Diptera (**Muscidae)	**Multiple, 5–11 × 0.3–1.5**	**Laos**	**Ovoid to obclavate, 380 –540 × 150–220**	**Cylindrical, 430–610 × 5.8–7.0**	**—**	**This study**
*O. muscidarum*	Diptera (Muscidae)	Single or Multiple, 5–7 × 1–4	China	flask-shaped, 570–760 × 190–310	Cylindrical,280–430 × 5.4–7.5	Fusiform, 7–10.5 × 1.6–2.5	Yang et al. 2025
***O. muscidarum***	**Diptera (Muscidae)**	**Multiple, 2–3 × 0.4–1.7**	**China**	**flask-shaped, 600–780 × 130–240**	**Cylindrical, 310–400 × 4.2–6.8**	**Cylindrical, 7.2–9.5 × 1.0–2.6**	**This study**

#### Ophiocordyceps laosensis

Taxon classificationFungiOphiocordycipitaceae

Y. Wang & Y.H. Guan sp. nov.

28CC8F80-90E8-5EFA-B839-60145F24EC9D

860784

Fig. 3

##### Etymology.

The epithet refers to the country (Laos) where the type specimen was collected.

##### Holotype.

Laos, • Oudomxay Province, Muang Xay City (20.26°N, 101.38°E, alt. 1032 m), parasitic on an adult of the housefly (*Musca* sp.), collected on leaves, 6 Aug 2024, Yao Wang (GMB 3137).

##### Description.

**Figure 3.**
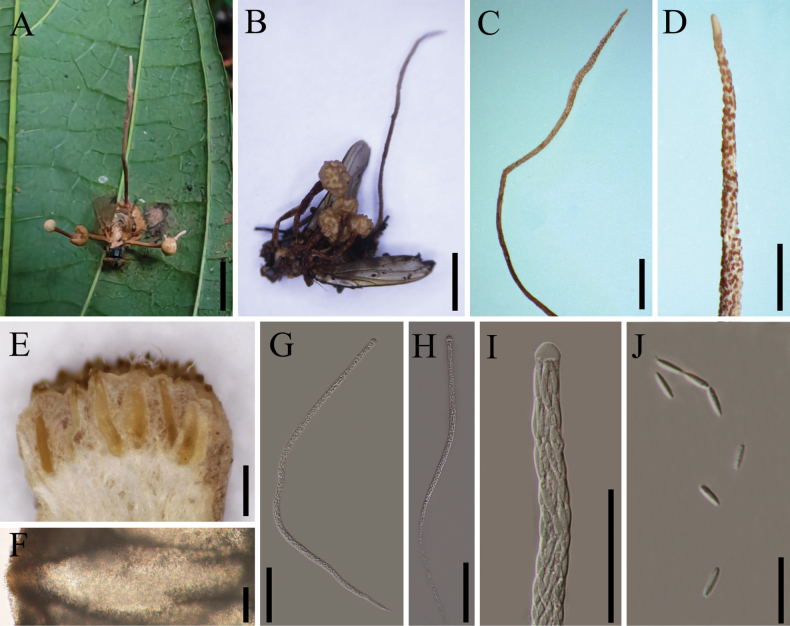
*Ophiocordyceps laosensis*. **A, B**. Fungus on housefly (*Musca* sp., Muscidae, Diptera); **C, D**. Columnar stromata bearing wart-like protuberances; **E, F**. Perithecia; **G, H**. Asci; **I, J**. Part-spores. Scale bars: 5 mm (**A, B**); 1 mm (**C, D**); 500 µm (**E**); 100 µm (**F–H**); 50 µm (**I**); 20 µm (**J**).

**Sexual morph**: ***Stromata*** stipitate, one or several arising from the thorax region of the host, beneath the wings, capitate, unbranched, brown, 5–9 mm long, 0.5–1.3 mm wide with a fertile apex (Fig. 3A, B). ***Fertile heads*** globoid, surface convex, Orange-yellow to brown, located at the terminal part of stipes, 0.8–1.7 mm thick, 1.6–3.8 mm diam (Fig. 3B). ***Perithecia*** 320–1300 × 150–380 μm (x̄= 864 × 283 µm, n = 20), immersed, flask-shaped. ***Asci*** 480–570 × 4.0–12.0 μm (x̄ = 516 × 9.2 µm, n = 20), 8-spored, hyaline, cylindrical. ***Apical cap*** 4.2–8.5 × 5.8–11.7 μm (x̄ = 6.4 × 9.3 µm, n = 20), thick, hyaline. ***Ascospores*** filiform, multi-septate, breaking into many (~8) part-spores, cylindrical, 11–15 × 2.0–4.7 μm (x̄ = 13.2 × 3.5 µm, n = 20). **Asexual morph**: Not observed in natural substrates.

##### Host.

*Musca* sp. (Muscidae, Diptera).

##### Habitat.

The specimens were found on the underside of a dicotyledonous leaf from a forest plant.

##### Other material examined.

Laos, • Oudomxay Province, Muang Xay City (20.12°N, 101.06°E, alt. 986 m), parasitic on an adult of *Musca* sp. (Muscidae, Diptera), collected on leaves, 7 Aug 2024, Yao Wang (GMB 3138).

##### Notes.

*Ophiocordyceps laosensis* possesses two distinct types of stromata: globose and columnar. The globose stromata contain developing asci and ascospores, whereas the columnar stromata bear wart-like projections on the surface, which may superficially resemble asci but are, in fact, non-fertile structures.

Phylogenetic analyses revealed that *O. laosensis* formed a distinct lineage within the *O. muscae* core group, with strong statistical support (Fig. 1; 100%/100%/1). *Ophiocordyceps laosensis* closely resembles *O. muscae*, as both species parasitize dipteran hosts. Both species produce rod-shaped stromata capped by fertile heads with nearly spherical surface projections. However, *O. laosensis* can be distinguished from *O. muscae* by its more elongated perithecial ostioles, as well as consistently larger asci and part-spores (Table 2).

#### Ophiocordyceps muscae

Taxon classificationFungiOphiocordycipitaceae

Mongkolsamrit, Liangsiri, Thanakitpipattana & Luangsa-ard, MycoKeys 119: 244 (2025)

51D1E11C-6922-58F4-A8A5-3F529845B55A

858733

Fig. 4

##### Note.

The description and illustrations were based on specimens of *O. muscae* collected in Laos.

##### Description.

**Figure 4.**
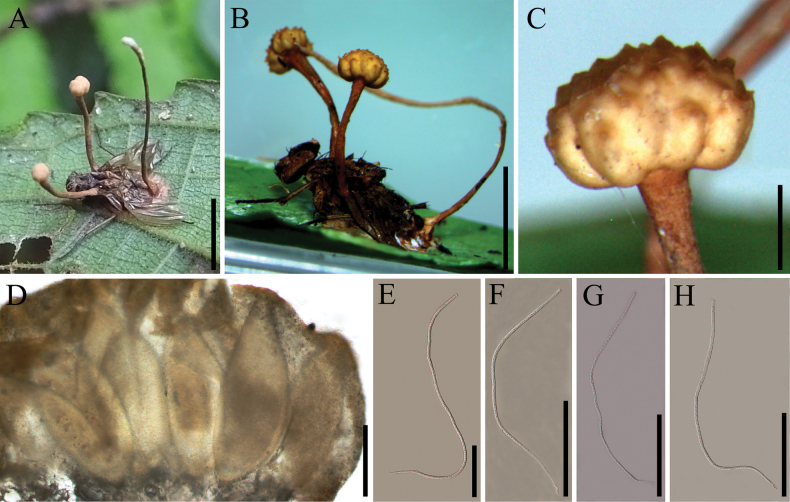
*Ophiocordyceps muscae*. **A, B**. Fungus on housefly (*Musca domestica*, Muscidae, Diptera); **C**. Stromata; **D**. Perithecia; **E–H**. Asci. Scale bars: 5 mm (**A, B**); 1 mm (**C**); 200 µm (**D–H**).

**Sexual morph**: Stromata 5–11 mm long, 0.3–1.5 mm wide, multiple, stipitate, cylindrical, capitate, pale yellow to brown, arising from the thorax region of the host (Fig. 4A, B). ***Fertile heads*** 1.0–1.5 × 1.4–2.6 mm, dark brown, with asexual morph at the apex (Fig. 4B, C). ***Perithecia*** 380–540 × 150–220 μm (x̄= 430 × 176 µm, n = 20), immersed, ovoid to obclavate. ***Asci*** 430–610 × 5.8–7.0 μm (x̄ = 554 × 6.3 µm, n = 20), 8-spored, hyaline, cylindrical. ***Apical cap*** 3.2–5.7 × 5.5–10.3 μm (x̄ = 4.6 × 8.2 µm, n = 20), thick, hyaline. **Asexual morph**: Not observed in natural substrates.

##### Materials examined.

Laos, • Oudomxay Province, Namkat Yolapa Resort (19.93°N, 100.36°E, alt. 1069 m), parasitic on *Musca* sp. (Muscidae, Diptera) on the leaves, 6 Aug 2025, Yao Wang (GMB 3135, GMB 3136).

##### Notes.

*Ophiocordyceps muscae* was first reported by Mongkolsamrit et al. (2025) based on specimens collected in Thailand. Phylogenetic analysis confirmed that the newly collected Laotian specimens (GMB 3135, GMB 3136) cluster within the *O. muscae* clade with high statistical support (Fig. 1; 100%/100%/1). Morphometric comparisons revealed that the perithecia of these specimens are smaller than those of the type material; however, no significant differences were observed in macroscopic morphology or other reproductive structures. Based on integrated molecular and morphological evidence, the newly collected specimens are identified as *O. muscae*. This collection represents the first documented occurrence of *O. muscae* in Laos.

#### Ophiocordyceps muscidarum

Taxon classificationFungiOphiocordycipitaceae

Y. P. Xiao, K.D. Hyde & Y. Yang, MycoKeys 117: 298 (2025)

C4F98F28-8298-5510-B321-50FAA78AFDE6

902879

Index Fungorum: IF902879

Facesoffungi Number: FoF16766

Fig. 5

##### Note.

The description and illustrations are based on specimens of *O. muscidarum* collected during this study.

##### Description.

**Figure 5.**
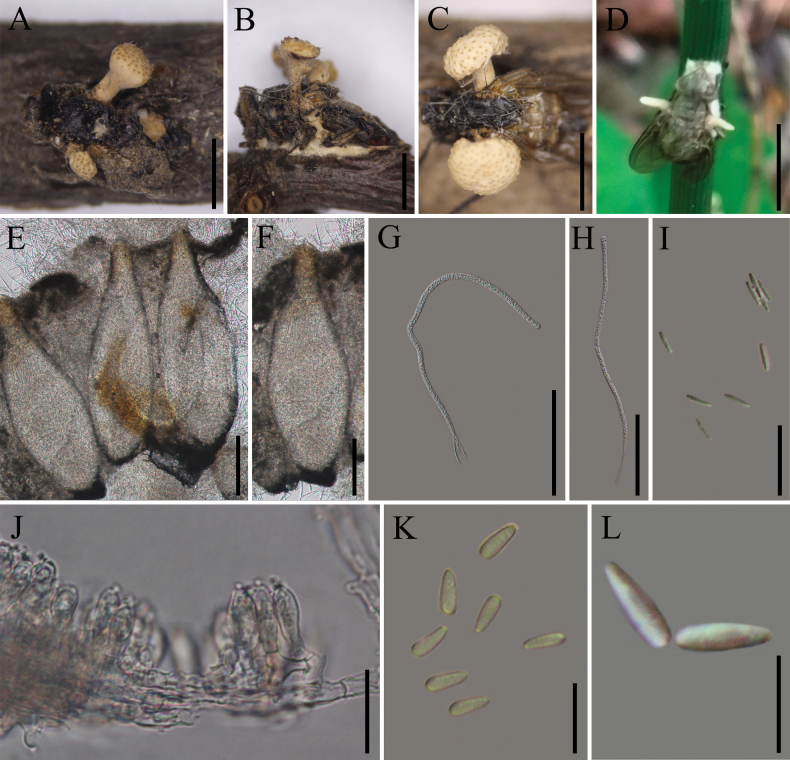
*Ophiocordyceps muscidarum*. **A, B**. Fungus on fly (Muscidae, Diptera); **C**. Stromata; **D**. The early-stage infected host by the *Ophiocordyceps* fungus; **E, F**. Perithecia; **G, H**. Asci; **I**. Part-spores; **J**. Phialides; **K, L**. Conidia. Scale bars: 2 mm (**A, B**); 5 mm (**C, D**); 200 µm (**E, F**); 100 µm (**G, H**); 20 µm (**I, J**); 10 µm (**K**); 5 µm (**L**).

**Sexual morph**: Stromata 2–3 mm long, 0.4–1.7 mm wide, multiple, stipitate, cylindrical, capitate, pale yellow, arising from both sides of the host’s body (Fig. 5A–D). ***Fertile heads*** 1.4–2.2 × 2.6–4.8 mm, discoid, pale yellow, with asexual morph at the apex (Fig. 5C). ***Perithecia*** 600–780 × 130–240 μm (x̄= 683 × 194 µm, n = 20), immersed, bowling-pin-shaped, ovoid to obclavate. ***Asci*** 310–400 × 4.2–6.8 μm (x̄ = 346 ×5.3 µm, n = 50), 8-spored, hyaline, cylindrical. ***Apical cap*** 4.8–8.2 × 2.8–4.3 μm (x̄ = 6.5 × 3.4µm, n = 50), thick, hyaline. **Asexual morph**: Asexual spore-producing structures were observed on the host’s hind limbs. ***Conidiogenous cells****Hymenostilbe*-like, phialidic, forming a hymenial layer. ***Phialides*** single, 8.6–16.5 × 3.5–7.4 µm (x̄ = 13.6 × 5.7µm, n = 20). Middle portion strongly swollen, usually tapering abruptly to a slender neck 0.3–1.0 µm diam. ***Conidia*** 3.6–6.8 × 1.5– 3.1 µm (x̄ = 5.1 × 2.6 µm, n = 20), 1-cell, hyaline, fusiform.

##### Materials examined.

China. • Heilongjiang Province, Yichun City (48.13°N, 129.61°E, alt. 832 m), on *Coenosia* sp., 23 July 2025, collected by Kun Zhang (GMB 3132, GMB 3133). China. • Jilin Province, Dunhua City (44.12°N, 128.63°E, alt. 596 m), on *Helina* sp., Aug 2025, collected by Kun Zhang (GMB 3134). China. • Jilin Province, Dunhua City (43.62°N, 127.54°E, alt. 610 m), on an adult muscid fly (Diptera: Muscidae), Aug 2025, collected by Kun Zhang (GMB 3141).

##### Notes.

*Ophiocordyceps muscidarum* was first described by Yang et al. (2025). Phylogenetic analyses showed that our newly collected specimens (GMB 3132, GMB 3133) clustered within the *O. muscidarum* clade with strong support (Fig. 1; 99/99/1), exhibiting no genetic differentiation. Morphological comparisons revealed no notable differences, except that the stromata observed in our specimens were slightly shorter. Based on the combined molecular and morphological evidence, the specimens were identified as *O. muscidarum*. Furthermore, as the original description lacked information on the asexual stage of this species, partial asexual morphological data for *O. muscidarum* was provided here based on our specimen observations.

## Discussion

A systematic taxonomic study was conducted on dipteran-parasitizing *Ophiocordyceps* species based on morphological comparison and a concatenated dataset containing five loci (ITS, *nr*LSU, *tef-1α*, *rpb1*, and *rpb2*) phylogenetic analyses. One of the new species, *O. calliphoridarum*, is closely related to *O. muscidarum* but differs by parasitizing *Lucilia caesar* (Calliphoridae) rather than the housefly (Muscidae) and by possessing significantly larger asci and part-spores. While *O. laosensis* closely resembles *O. muscae*, it can be distinguished by its elongated perithecial ostioles, as well as its large asci and part-spores. These two newly described species, together with the ten previously reported dipteran-parasitizing *Ophiocordyceps* species, formed a well-supported monophyletic clade—the *O. dipterigena* complex (Mongkolsamrit et al. 2025), providing compelling evidence for the monophyly of *Ophiocordyceps* species infecting dipteran hosts. However, *O. forquignonii* represents an exception, as it parasitizes Diptera but falls outside this clade, being positioned at the terminal of the hymenostilboid clade. Our findings contribute to a better understanding and expansion of the species diversity of this complex. It should be noted that *Ophiocordyceps lacrimoidis* was previously considered part of the *O. dipterigena* complex (Hyde et al. 2016; Mongkolsamrit et al. 2025). However, its phylogenetic position is currently inferred solely from the ITS sequence (Hyde et al. 2016), and based on our analyses, it does not cluster within the *O. dipterigena* complex clade (Suppl. material 1). Although it shares morphological and ecological similarities with members of this complex—being parasitic on Muscidae, possessing a capitated sexual structure with a discoid fertile head, and exhibiting a *Hymenostilbe*-like asexual morph (Hyde et al. 2016), its phylogenetic placement still requires reassessment using additional molecular markers.

Although we have documented morphological differences among the species within *O. dipterigena* complex, these taxa show pronounced considerable morphological convergence at the macroscopic level, typically characterized by pale yellow to brown, clavate stromata bearing fertile ascomata at their apex. Consequently, four species within this clade—*O. dipterigena*, *O. discoideicapitata* (Kobayasi and Shimizu 1982), *Cordyceps muscicola* (Möller 1901; Freire 2015) and *Cordyceps sakishimensis* (Kobayasi and Shimizu 1983), had been reported prior to the application of molecular-assisted identification. This study underscores the critical role of multi-gene data in the species identification, delimitation and systematic phylogeny of cryptic species within this species complex. By conducting five-locus phylogenetic analyses (ITS, *nr*LSU, *tef-1α*, *rpb1*, and *rpb2*), we identified two new species within the *O. dipterigena* complex, as well as a new record from Laos, thereby extending the known geographic distribution, and providing additional biogeographical evidence for the diversity of this group in southeast Asia. The results not only refine the taxonomic framework of the *O. dipterigena* complex but also lay an important foundation for further investigations into the coevolutionary dynamics and ecological adaptations of *Ophiocordyceps* species and their dipteran hosts.

Notably, all specimens of *O. laosensis* and *O. muscae* from Laos were found on the underside of leaves, whereas most *O. calliphoridarum* and *O. muscidarum* specimens from temperate regions of China occurred on stems. Consistent with prior records, tropical members of the *O. dipterigena* complex, including *O. muscae*, *O. floriformis*, and *O. thilosuensis* from Thailand, predominantly inhabit abaxial leaf surfaces (Mongkolsamrit et al. 2025). This distinct microhabitat differentiation is likely influenced by climatic factors: the high temperatures and heavy rainfall of tropical regions may favor development on sheltered leaf undersides, which offer higher humidity and protection from sunlight and precipitation. We suggest that this pattern is not the result of targeted host behavior manipulation, but rather represents an adaptive response to the combined effects of environmental conditions, such as temperature, humidity, and rainfall. Nonetheless, broader sampling and further studies are required to clarify the ecological mechanisms underlying these habitat preferences.

## Supplementary Material

XML Treatment for Ophiocordyceps calliphoridarum

XML Treatment for Ophiocordyceps laosensis

XML Treatment for Ophiocordyceps muscae

XML Treatment for Ophiocordyceps muscidarum

## Additional information

### Use of AI

The authors did not use Artificial Intelligence (AI) or AI-assisted tools during the preparation of my manuscript.

### Author ORCIDs

Yong-dong Dai https://orcid.org/0000-0001-8262-9803

Yu-Hu Guan https://orcid.org/0009-0006-5764-8205

Hui Chen https://orcid.org/0009-0008-0291-3571

Chanhom Loinheuang https://orcid.org/0009-0008-4852-9700

Jiandong Liang https://orcid.org/0000-0002-3939-3900

Yao Wang https://orcid.org/0000-0002-1262-6700

### Data availability

All of the data that support the findings of this study are available in the main text. All sequences were submitted to GenBank and obtained the accession number.

## Supplementary materials

Supplementary material 1 Phylogenetic tree of *Ophiocordyceps* with the ITS sequences Data type docx Explanation note *Ophiocordyceps lacrimoidis* was previously placed within *O. dipterigena* complex (Mongkolsamrit et al. 2025). However, this species was represented only by the ITS sequence, in our phylogenetic reconstruction, it did not cluster within *O. dipterigena* complex. https://binary.pensoft.net/file/1518924This dataset is made available under the Open Database License (http://opendatacommons.org/licenses/odbl/1.0/). The Open Database License (ODbL) is a license agreement intended to allow users to freely share, modify, and use this Dataset while maintaining this same freedom for others, provided that the original source and author(s) are credited. Yong-dong Dai, Yu-Hu Guan, Sheng-mei Wu, Shabana Bibi, Hui Chen, Chanhom Loinheuang, Jiandong Liang, Yao Wang

## References

[B1] Araújo JP, Hughes DP (2019) Zombie-ant fungi emerged from non-manipulating, beetle-infecting ancestors. Current Biology 29(21): 3735–3738. 10.1016/j.cub.2019.09.004 31668622

[B2] Araújo JPM, Evans HC, Kepler R, Hughes DP (2018) Zombie-ant fungi across continents: 15 new species and new combinations within *Ophiocordyceps*. I. myrmecophilous hirsutelloid species. Studies in Mycology 90(1): 119–160. 10.1016/j.simyco.2017.12.002 PMC6002356 29910522

[B3] Ban S, Sakane T, Nakagiri A (2015) Three new species of *Ophiocordyceps* and overview of anamorph types in the genus and the family Ophiocordycipitaceae. Mycological Progress 14: 1017–1028. 10.1007/s11557-014-1017-8

[B4] Berkeley MJ, Broome CE (1873) Enumeration of the Fungi of Ceylon. Part II., containing the remainder of the Hymenomycetes, with the remaining established tribes of Fungi. Botanical Journal of the Linnean Society 14(74): 29–140. 10.1111/j.1095-8339.1873.tb00302.x

[B5] Castlebury LA, Rossman AY, Sung GH, Hyten AS, Spatafora JW (2004) Multigene phylogeny reveals new lineage for Stachybotrys chartarum, the indoor air fungus. Mycological Research 108(8): 864–872. 10.1017/S0953756204000607 15449591

[B6] Chang CX, Zhang ZX, Deng LP, Liang JD, Yu Q, Dai YD (2026) *Ophiocordyceps paraisarioidea* sp. nov. (Hypocreales, Ophiocordycipitaceae), a novel species of *Ophiocordyceps* parasitizing lepidopteran larvae. Mycosystema 45(1): е250213. 10.13346/j.mycosystema.250213

[B7] Chen J, Li W, Mi Q, Zhang F, Shi S, Zhang J (2020) A newly reported parasitoid, *Pentatomophaga latifascia* (Diptera: Tachinidae), of adult *Halyomorpha halys* in Beijing, China. Insects 11(10): е666. 10.3390/insects11100666 PMC7601123 33003332

[B8] Crous PW, Costa MM, Kandemir H, Vermaas M, Vu D, Zhao L, Arumugam E, Flakus A, Jurjević Ž, Kaliyaperumal M (2023) Fungal Planet description sheets: 1550–1613. Persoonia 51: 280–417. 10.3767/persoonia.2023.51.08 PMC11041897 38665977

[B9] Crous PW, Luangsa-ard JJ, Wingfield MJ, Carnegie AJ, Hernández-Restrepo M, Lombard L, Roux J, Barreto RW, Baseia IG, Cano-Lira JF (2018) Fungal Planet description sheets: 785–867. Persoonia 41: 238–417. 10.3767/persoonia.2018.41.12 PMC6344811 30728607

[B10] Dai YD, Chen SQ, Wang YB, Wang Y, Yang ZL, Yu H (2024) Molecular phylogenetics of the *Ophiocordyceps sinensis*-species complex lineage (Ascomycota, Hypocreales), with the discovery of new species and predictions of species distribution. IMA Fungus 15: 1–2. 10.1186/s43008-023-00131-8 PMC10858606 38336758

[B11] Dong CH, Liu XZ, Li ZZ, Yan WJ, Li TH (2016) *Cordyceps* industry in China: Current status, challenges and perspectives —Jinhu declaration for *Cordyceps* industry development. Mycosystema 35(1): 1–15. 10.13346/j.mycosystema.150207

[B12] Chen H, Bibi S, Tao L, Shen XC, Zhao J, Sun YM, Li QR, Tang DX, Wang Y (2025) Papiliomyces sinensis (Clavicipitaceae) and Paraisaria pseudoarcta (Ophiocordycipitaceae), two new species parasitizing Lepidopteran insects from southwestern China. MycoKeys 17: 353–374. 10.3897/mycokeys.117.150376 PMC12099317 40417394

[B13] Evans HC, Elliot SL, Hughes D (2011) *Ophiocordyceps unilateralis*: A keystone species for unraveling ecosystem functioning and biodiversity of fungi in tropical forests? Communicative & Integrative Biology 4: 598–602. 10.4161/cib.16721 PMC3204140 22046474

[B14] Folmer O, Black M, Hoeh W, Lutz R, Vrijenhoek R (1994) DNA primers for amplification of mitochondrial cytochrome c oxidase subunit I from diverse metazoan invertebrates. Molecular Marine Biology and Biotechnology 3(5): 294–299. 7881515

[B15] Freire FM (2015) Taxonomia e Distribuição de *Ophiocordyceps dipterigena* (Ophiocordy cipitaceae, Hypocreales). Repositório Institucional da UFSC, 128 pp.

[B16] Friedrich RCS, Shrestha B, Salvador-Montoya C, Tomé LM, Reck M, Góes-Neto A, Drechsler-Santos E (2018) *Ophiocordyceps neonutans* sp. nov., a new neotropical species from *O. nutans* complex (Ophiocordycipitaceae, Ascomycota). Phytotaxa 344(3): 215–227. 10.11646/phytotaxa.344.3.2

[B17] Guan YH, Chen H, Ren YL, Zhou ZQ, Zhang X, Wang Y (2025) *Ophiocordyceps anshunensis* sp. nov., a new entomopathogenic fungus from *O. nutans* complex (Ophiocordycipitaceae, Ascomycota). Phytotaxa 696: 132–144. 10.11646/phytotaxa.696.2.3

[B18] Hammami S, Ezzine O, Bystrowski C, Ben Jamâa ML (2023) A first detection of *Exorista segregata* (Rondani, 1859) (Diptera: Tachinidae) as a larval parasitoid of *Orgyia trigotephras* Boisduval, 1829 (Lepidoptera: Erebidae) from Tunisia. Egyptian Journal of Biological Pest Control 33(1): 1–9. 10.1186/s41938-023-00656-5

[B19] Hudiwaku S, Himawan T, Rizali A (2021) Diversity and species composition of fruit flies (Diptera: Tephritidae) in Lombok Island, Indonesia. Biodiversitas 22: 4608–4616. 10.13057/biodiv/d221054

[B20] Hyde KD, Chaiwan N, Norphanphoun C, Boonmee S, Camporesi E, Chethana KWT, Dayarathne MC, de Silva NI, Dissanayake AJ, Ekanayaka AH (2018) Mycosphere notes 169–224. Mycosphere 9(2): 271–430. 10.5943/mycosphere/9/2/8

[B21] Hyde KD, Hongsanan S, Jeewon R, Bhat DJ, McKenzie EHC, Jones EBG, Phookamsak R, Ariyawansa HA, Boonmee S, Zhao Q (2016) Fungal diversity notes 367–490: Taxonomic and phylogenetic contributions to fungal taxa. Fungal Diversity 80(1): 1–270. 10.1007/s13225-016-0373-x

[B22] Khonsanit A, Luangsa-Ard JJ, Thanakitpipattana D, Kobmoo N, Piasai O (2019) Cryptic species within *Ophiocordyceps myrmecophila* complex on formicine ants from Thailand. Mycological Progress 18: 147–161. 10.1007/s11557-018-1412-7

[B23] Khao-ngam S, Mongkolsamrit S, Rungjindamai N, Noisripoom W, Pooissarakul W, Duangthisan J, Himaman W, Luangsa-ard JJ (2021) *Ophiocordyceps asiana* and *Ophiocordyceps tessaratomidarum* (Ophiocordycipitaceae, Hypocreales), two new species on stink bugs from Thailand. Mycological Progress 20(3): 341–353. 10.1007/s11557-021-01684-x

[B24] Kobayasi Y, Shimizu D (1982) *Cordyceps* species from Japan. Bulletin of the National Science Museum 8(3): 79–91.

[B25] Kobayasi Y, Shimizu D (1983) Cordyceps species from Japan. 6. Bulletin of the National Science Museum Tokyo 9: 1–21.

[B26] Lanfear R, Frandsen PB, Wright AM, Senfeld T, Calcott B (2017) PartitionFinder 2: New methods for selecting partitioned models of evolution for molecular and morphological phylogenetic analyses. Molecular Biology and Evolution 34(3): 772–773. 10.1093/molbev/msw260 28013191

[B27] Liang ZQ (2007) Flora fungorun sinicorun (Vol. 32). *Cordyceps*. Science Press, Beijing, 190 pp.

[B28] Liu YJ, Whelen S, Hall BD (1999) Phylogenetic relationships among ascomycetes: Evidence from an RNA polymerse II subunit. Molecular Biology and Evolution 16(12): 1799–1808. 10.1093/oxfordjournals.molbev.a026092 10605121

[B29] Long FY, Qin LW, Xiao YP, Hyde KD, Wang SX, Wen TC (2021) Multigene phylogeny and morphology reveal a new species, *Ophiocordyceps vespulae*, from jilin province, China. Phytotaxa 478(1): 33–48. 10.11646/phytotaxa.478.1.2

[B30] Luangsa-ard JJ, Tasanathai K, Thanakitpipattana D, Khonsanit A, Stadler M (2018) Novel and interesting *Ophiocordyceps* spp. (Ophiocordycipitaceae, Hypocreales) with superficial perithecia from Thailand. Studies in Mycology 89: 125–142. 10.1016/j.simyco.2018.02.001 PMC6002337 29910519

[B31] Luangsa-Ard JJ, Ridkaew R, Tasanathai K, Thanakitpipattana D, Hywel-Jones N (2011) *Ophiocordyceps halabalaensis*: a new species of Ophiocordyceps pathogenic to *Camponotus gigas* in Hala Bala Wildlife Sanctuary, Southern Thailand. Fungal Biology 115(7): 608–614. 10.1016/j.funbio.2011.03.002 21724166

[B32] Martins C, Afonso C, Valente C, Reis AR, O’Hara J, Lumbers J, Branco M, Gonçalves CI (2023) *Anagonia lasiophthalma* (Diptera: Tachinidae): Survey, identification, and biological traits of a new biological control agent of the *Eucalyptus* snout beetle, *Gonipterus platensis* (Coleoptera: Curculionidae). Biological Control 187: e105391. 10.1016/j.biocontrol.2023.105391

[B33] Minh BQ, Schmidt HA, Chernomor O, Schrempf D, Woodhams MD, Von Haeseler A, Lanfear R (2020) IQ-TREE 2: New models and efficient methods for phylogenetic inference in the genomic era. Molecular Biology and Evolution 37: 1530–1534. 10.1093/molbev/msaa015 PMC7182206 32011700

[B34] Möller A (1901) Phycomyceten und Ascomyceten. Untersuchungen aus Brasilien. Botanische Mittheilungen aus den Tropen 9: 1–319. 10.5962/bhl.title.31997

[B35] Mongkolsamrit S, Noisripoom W, Pumiputikul S, Boonlarppradab C, Stadler M, Becke K, Luangsa-Ard JJ (2021) *Ophiocordyceps flavida* sp. nov. (Ophiocordycipitaceae), a new species from Thailand associated with *Pseudogibellula formicarum* (Cordycipitaceae), and their bioactive secondary metabolites. Mycological Progress 20: 477–492. 10.1007/s11557-021-01683-y

[B36] Mongkolsamrit S, Noisripoom W, Tasanathai K, Khonsanit A, Thanakitpipattana D, Lamlertthon S, Himaman W, Crous PW, Stadler M, Luangsa-Ard JJ (2024) Uncovering cryptic species diversity of *Ophiocordyceps* (Ophiocordycipitaceae) associated with Coleoptera from Thailand. Fungal Systematics and Evolution 14: 223–250. 10.3114/fuse.2024.14.15 PMC11736259 39830293

[B37] Mongkolsamrit S, Noisripoom W, Arnamnart N, Lamlertthon S, Himaman W, Jangsantear P, Samson RA, Luangsa-ard JJ (2019) Resurrection of Paraisaria in the Ophiocordycipitaceae with three new species from Thailand. Mycological Progress 18: 1213–1230. 10.1007/s11557-019-01518-x

[B38] Mongkolsamrit S, Thanakitpipattana D, Noisripoom W, Tasanathai K, Liangsiri K, Jaiyen S, Rungjindamai N, Stadler M, Luangsa-ard JJ (2025) Multi-locus molecular phylogenetic analysis reveals four new species and a new record of *Ophiocordyceps* (Ophiocordycipitaceae, Hypocreales) on dipteran hosts in Thailand. MycoKeys 119: 235–261. 10.3897/mycokeys.119.155439 PMC12260605 40666087

[B39] Nainu F, Salim E, Emran TB, Sharma R (2022) *Drosophila melanogaster* as a versatile model for studying medically important insect vector-borne parasites. Frontiers in Cellular and Infection Microbiology 12: e939813. 10.3389/fcimb.2022.939813 PMC9201246 35719344

[B40] Quandt CA, Kepler RM, Gams W, Araújo JPM, Ban S, Evans HC, Hughes D, Humber R, Hywel-Jones NL, Li ZZ, Luangsa-ard JJ, Rehner SA, Sanjuan T, Sato H, Shrestha B, Sung GH, Yao YJ, Zare R, Spatafora JW (2014) Phylogenetic-based nomenclatural proposals for Ophiocordycipitaceae (Hypocreales) with new combinations in *Tolypocladium*. IMA Fungus 5(1): 121–134. 10.5598/imafungus.2014.05.01.12 PMC4107890 25083412

[B41] Rath AC (2000) The use of entomopathogenic fungi for control of termites. Biocontrol Science and Technology 10(5): 563–581. 10.1080/095831500750016370

[B42] Rehner SA, Buckley E (2005) A *Beauveria* phylogeny inferred from nuclear ITS and EF1 α sequences: Evidence for cryptic diversification and links to *Cordyceps* teleomorphs. Mycologia 97(1): 84–98. 10.3852/mycologia.97.1.84 16389960

[B43] Ronquist F, Teslenko M, Van Der Mark P, Ayres DL, Darling A, Höhna S, Larget B, Liu L, Suchard MA, Huelsenbeck JP (2012) MrBayes 3.2: Efficient Bayesian phylogenetic inference and model choice across a large model space. Systematic Biology 61: 539–542. 10.1093/sysbio/sys029 PMC3329765 22357727

[B44] Salgado-Neto G, Sanz‐Veiga PA, Vaz MAB (2018) First record of *Ophiocordyceps dipterigena* (Ascomycota: Hypocreales: Ophiocordycipitaceae) infecting adults of *Melanagromyza sojae* (Diptera: Agromyzidae) in Brazil. Ciência Rural 48(7): e20170637. 10.1590/0103-8478cr20170637

[B45] Sanjuan T, Francomolano AE, Kepler RM, Spatafora JW, Tabima J, Vasco-Palacios AM, Restrepo S (2015) Five new species of entomopathogenic fungi from the Amazon and evolution of neotropical *Ophiocordyceps*. Fungal Biology 119(10): 901–916. 10.1016/j.funbio.2015.06.010 26399185

[B46] Sasaki F, Miyamoto T, Yamamoto A, Tamai Y, Yajima T (2008) Morphological and genetic characteristics of the entomopathogenic fungus *Ophiocordyceps nutans* and its host insects. Mycological Research 112: 1241–1244. 10.1016/j.mycres.2008.04.008 18693103

[B47] Schoch CL, Seifert KA, Huhndorf S, Robert V, Spouge JL, Levesque CA, Chen W, Fungal barcoding Consortium (2012) The nuclear ribosomal internal transcribed spacer (ITS) region as a universal DNA barcode marker for Fungi. Proceedings of the National Academy of Sciences of the United States of America 109: 6241–6246. 10.1073/pnas.1117018109 PMC3341068 22454494

[B48] Scolari F, Valerio F, Benelli G, Papadopoulos NT, Vaníčková L (2021) Tephritid fruit fly semiochemicals: Current knowledge and future perspectives. Insects 12(5): e408. 10.3390/insects12050408 PMC8147262 33946603

[B49] Shrestha B, Tanaka E, Hyun MW, Han JG, Kim CS, Jo JW, Han SK, Oh J, Sung GH (2016) Coleopteran and lepidopteran hosts of the entomopathogenic genus *Cordyceps* sensu lato. Journal of Mycology 2016: 1–14. 10.1155/2016/7648219

[B50] Spatafora JW, Sung GH, Sung JM, Hywel-Jones NL, White Jr JF (2007) Phylogenetic evidence for an animal pathogen origin of ergot and the grass endophytes. Molecular Ecology 16: 1701–1711. 10.1111/j.1365-294X.2007.03225.x 17402984

[B51] Stamatakis A (2014) RAxML version 8: A tool for phylogenetic analysis and post-analysis of large phylogenies. Bioinformatics 30(9): 1312–1313. 10.1093/bioinformatics/btu033 PMC3998144 24451623

[B52] Sung GH, Hywel-Jones NL, Sung JM, Luangsa-Ard JJ, Shrestha B, Spatafora JW (2007) Phylogenetic classification of *Cordyceps* and the clavicipitaceous fungi. Studies in Mycology 57: 5–59. 10.3114/sim.2007.57.01 PMC2104736 18490993

[B53] Tamura K, Stecher G, Peterson D, Filipski A, Kumar S (2013) MEGA6: Molecular evolutionary genetics analysis version 6.0. Molecular Biology and Evolution 30: 2725–2729. 10.1093/molbev/mst197 PMC3840312 24132122

[B54] Tang DX, Huang O, Zou WQ, Wang YB, Wang Y, Dong QY, Sun T, Yang G, Yu H (2023) Six new species of zombie-ant fungi from Yunnan in China. IMA Fungus 14(1): 1–9. 10.1186/s43008-023-00114-9 PMC10173673 37170179

[B55] Tasanathai K, Noisripoom W, Chaitika T, Khonsanit A, Hasin S, Luangsa-ard JJ (2019) Phylogenetic and morphological classification of *Ophiocordyceps* species on termites from Thailand. MycoKeys 56: 101–129. 10.3897/mycokeys.56.37636 PMC6684523 31402842

[B56] Tasanathai K, Khonsanit A, Noisripoom W, Kobmoo N, Luangsa-ard J (2022) Hidden species behind *Ophiocordyceps* (Ophiocordycipitaceae, Hypocreales) on termites: Four new species from Thailand. Mycological Progress 21(10): 1–86. 10.1007/s11557-022-01837-6

[B57] Vilgalys R, Hester M (1990) Rapid genetic identification and mapping of enzymatically amplified ribosomal DNA from several *Cryptococcus* species. Journal of Bacteriology 172(8): 4238–4246. 10.1128/jb.172.8.4238-4246.1990 PMC213247 2376561

[B58] Wang Y, Dai YD, Yang ZL, Guo R, Wang YB, Yang ZL, Ding L, Yu H (2021) Morphological and molecular phylogenetic data of the chinese medicinal fungus *Cordyceps liangshanensis* reveal its new systematic position in the family Ophiocordycipitaceae. Mycobiology 49(4): 297–307. 10.1080/12298093.2021.1923388 PMC8409936 34512076

[B59] Wang YB, Nguyen TT, Dai YD, Yu H, Zeng WB, Wu CK (2018) Molecular phylogeny and morphology of *Ophiocordyceps unituberculata* sp. nov. (Ophiocordycipitaceae), a pathogen of caterpillars (Noctuidae, Lepidoptera) from Yunnan, China. Mycological Progress 17: 745–753. 10.1007/s11557-017-1370-5

[B60] Wei DP, Gentekaki E, Wanasinghe DN, Tang SM, Hyde KD (2022) Diversity, molecular dating and ancestral characters state reconstruction of entomopathogenic fungi in Hypocreales. Mycosphere 13(2): 281–351. 10.5943/mycosphere/si/1f/8

[B61] Wei DP, Wanasinghe DN, Hyde KD, Mortimer PE, Xu JC, To-Anun C, Yu FM, Zha LS (2020) *Ophiocordyceps tianshanensis* sp. nov. on ants from Tianshan mountains, PR China. Phytotaxa 464(4): 277–292. 10.11646/phytotaxa.464.4.2

[B62] White TJ, Bruns T, Lee S, Taylor J (1990) Amplification and direct sequencing of fungal ribosomal RNA genes for phylogenetics. PCR Protocols: A guide to methods and applications 18: 315–322. 10.1016/B978-0-12-372180-8.50042-1

[B63] Wijayawardene NN, Hyde KD, Rajeshkumar KC, Hawksworth DL, Madrid H, Kirk PM, Braun U, Singh RV, Crous PW, Kukwa M, Luecking R (2017) Notes for genera: *Ascomycota*. Fungal Diversity 86: 1–594. 10.1007/s13225-017-0386-0

[B64] Will L, Das B, Trinh T, Brachmann A, Ohm RA, de Bekker C (2020) Genetic underpinnings of host manipulation by *Ophiocordyceps* as revealed by comparative transcriptomics. Genes Genomes Genetics 10: 2275–2296. 10.1534/g3.120.401290 PMC7341126 32354705

[B65] Wu F, Zhou LW, Yang ZL, Bau T, Li TH, Dai YC (2019) Resource diversity of Chinese macrofungi: Edible, medicinal and poisonous species. Fungal Diversity 98: 1–76. 10.1007/s13225-019-00432-7

[B66] Xiao YP, Wen TC, Hongsanan S, Sun JZ, Hyde KD (2017) Introducing *Ophiocordyceps thanathonensis*, a new species of entomogenous fungi on ants, and a reference specimen for *O. pseudolloydii*. Phytotaxa 328(2): e115. 10.11646/phytotaxa.328.2.2

[B67] Xiao YP, Hongsanan S, Hyde KD, Brooks S, Xie N, Long FY, Wen TC (2019) Two new entomopathogenic species of *Ophiocordyceps* in Thailand. MycoKeys 47: 53–74. 10.3897/mycokeys.47.29898 PMC6395454 30828254

[B68] Xu ZS, Deng LP, Wang HY, Tian HL, Qu JJ, Dai YD, Zou X (2025) Description of two new species of *Ophiocordyceps*: *O. sinocampes* and *O. cystidiata* (Ophiocordycipitaceae, Hypocreales) from typical karst landform forests in Guizhou, China. Mycokeys 114: 1–27. 10.3897/mycokeys.114.134323 PMC11843171 39990920

[B69] Yang Y, Xiao YP, Yu GJ, Wen TC, Deng CY, Meng J, Lu ZH (2021) *Ophiocordyceps aphrophoridarum* sp. nov., a new entomopathogenic species from Guizhou, China. Biodiversity Data Journal 9: e66115. 10.3897/BDJ.9.e66115 PMC8716513 34975278

[B70] Yang Y, Xiao YP, Jayawardena RS, Hyde KD, Nilthong S, Mapook A, Lu YZ, Xie SQ, Al-Otibi F, Wang X, Luo K, Luo LP (2025) Three new species of *Ophiocordyceps* (Hypocreales, Ophiocordycipitaceae) and a new host record for *Hirsutella vermicola* from China. MycoKeys 117: 289–313. 10.3897/mycokeys.117.144875 PMC12093112 40400764

[B71] Yu D, Chen L, Guo JL (2010) A review of research on *Cordyceps liangshanensis*. Journal of Li-shizhen Traditional Chinese Medicine 21(8): 2024–2025+2027.

