# Supplementary material for: Taxonomic and phylogenetic insights into dipteran-parasitizing Ophiocordyceps: Descriptions of two new species and a new record from China and Laos
Source: MycoKeys. 2026 Jan 23;127:217–38. doi: 10.3897/mycokeys.127.176148 (PMC12859645; doi:10.3897/mycokeys.127.176148)
Supplement: Supplementary material 1 — Phylogenetic tree of Ophiocordyceps with the ITS sequences [file mycokeys-127-217-s001.docx]

**Figure S1.** Phylogenetic tree of *Ophiocordyceps* based on Maximum Likelihood analysis from the ITS sequences. Numbers at the branches indicate support values (BI-PP) above 0.5. Isolates in bold type are those analyzed in this study.
